# Quality of spirometry and related diagnosis in primary care with a focus on clinical use

**DOI:** 10.1038/s41533-020-0177-z

**Published:** 2020-05-15

**Authors:** S. J. van de Hei, B. M. J. Flokstra-de Blok, H. J. Baretta, N. E. Doornewaard, T. van der Molen, K. W. Patberg, E. C. M. Ruberg, T. R. J. Schermer, I. Steenbruggen, J. W. K. van den Berg, J. W. H. Kocks

**Affiliations:** 1 Department of General Practice and Elderly Care Medicine, University of Groningen, University Medical Center Groningen, Groningen, the Netherlands; 2 Groningen Research Institute for Asthma and COPD (GRIAC), University of Groningen, University Medical Center Groningen, Groningen, the Netherlands; 3 Department of Pediatric Pulmonology and Pediatric Allergology, Beatrix Children’s Hospital, University of Groningen, University Medical Center Groningen, Groningen, the Netherlands; 4 General Practitioners Research Institute, Groningen, the Netherlands; 5 Department of Pulmonology, Isala Hospital, Zwolle, the Netherlands; 6 Pulmonary Laboratory, Isala, Zwolle, the Netherlands; 7 Department of Primary Community Care, Radboud University Medical Center, Nijmegen, the Netherlands

**Keywords:** Diagnosis, Respiratory tract diseases, Outcomes research

## Abstract

American and European societies’ (ATS/ERS) criteria for spirometry are often not met in primary care. Yet, it is unknown if quality is sufficient for daily clinical use. We evaluated quality of spirometry in primary care based on clinical usefulness, meeting ATS/ERS criteria and agreement on diagnosis between general practitioners (GPs) and pulmonologists. GPs included ten consecutive spirometry tests and detailed history questionnaires of patients who underwent spirometry as part of usual care. GPs and two pulmonologists assessed the spirometry tests and questionnaires on clinical usefulness and formulated a diagnosis. In total, 149 participants covering 15 GPs were included. Low agreements were found on diagnosis between GPs and pulmonologists 1 (*κ* = 0.39) and 2 (*κ* = 0.44). GPs and pulmonologists rated >88% of the tests as clinically useful, although 13% met ATS/ERS criteria. This real-life study demonstrated that clinical usefulness of routine primary care spirometry tests was high, although agreement on diagnosis was low.

## Introduction

Chronic airway diseases occur frequently, and it is estimated that more than 300 million people suffer from asthma worldwide and approximately 170 million people are affected by chronic obstructive pulmonary disease (COPD)^1^. Spirometry is essential for diagnosing airway obstruction and monitoring chronic respiratory diseases and is recommended in national and international guidelines^2–5^. Because most of the respiratory patients are diagnosed and managed by their general practitioner, spirometry is commonly used in primary care^6^. Performing spirometry in primary care lowers the burden for patients by preventing hospital visits, reduces the costs and provides quick results for the general practitioner (GP). In 2007, almost all Dutch General Practices had access to a spirometry facility, with two-third of the practices making use of their own spirometer^6^.

Good-quality spirometry requires reliable equipment, cooperation between a well-trained operator and a motivated patient, and an experienced interpreter^7^. Education demonstrated a positive effect on the quality of spirometry in primary care^8,9^. Furthermore, conducting spirometry frequently seems important to maintain the ability for accurate measurements^9^.

The quality of spirometry is traditionally assessed using the measures of acceptability and repeatability as formulated by the American Thoracic Society (ATS) and the European Respiratory Society (ERS)^7^, and has been investigated in primary care^9–12^. A Dutch study performed by Landman et al.^12^ demonstrated that 31.9% of spirometry tests in primary care practices and 60.3% of spirometry tests in primary care laboratories (where specialised lung function technicians conducted the tests) met the ATS/ERS criteria. However, 83.7 and 96.5% of the tests conducted in primary care practices and in primary care laboratories respectively were estimated to be clinically useful based on the opinion of experienced lung function technicians.

A different approach to quality of spirometry could be the quality being sufficient for daily clinical use when combined with structured clinical data. This approach of quality could be more relevant for good clinical care than criteria to assess the quality of the spirometry test itself. This study aimed to evaluate the quality of spirometry in primary care practices by agreement on respiratory diagnosis between general practitioners and pulmonologist in a real-life setting. In addition, this study aimed to assess the actual proportion of clinically useful spirometry tests in primary care based on the opinion of pulmonologists.

## Results

### General practices and participants

**Fig. 1:**
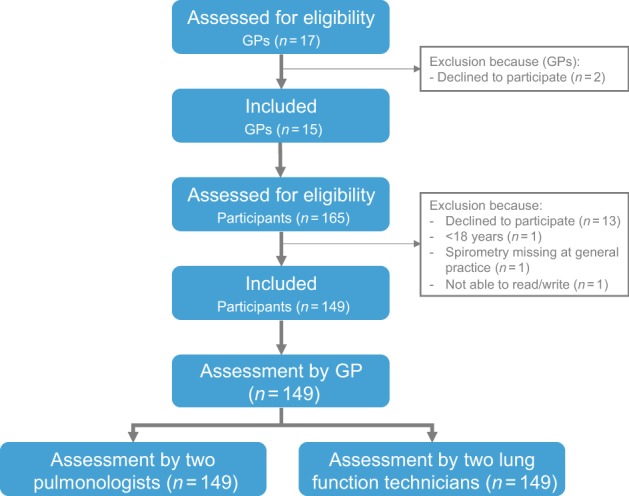
Flow of GPs and participants through the study. Flowchart.

This study was conducted between June 2017 and September 2018 in 13 general practices covering 15 GPs and 16 practice nurses. In total, 165 participants were screened for eligibility and 149 participants were included. Three practices only included nine spirometry tests and two practices included 11 tests. A flowchart of the study is shown in Fig. 1. The population consisted of 51.7% males, with a mean age 56.8 years (SD 17.2) and the mean FEV_1_ % predicted was 79.1% (SD 19.6%) (Table 1).

**Table 1:** Characteristics of participating general practices and participants.

General practices (*n* = 13)	
Annual number of spirometry tests performed (*n* = 12), *n* (%)	
≤40	0 (0.0)
41−80	4 (33.3)
81−120	4 (33.3)
>120	4 (33.3)
Participation in accredited spirometry educational programme, *n* (%)	
General practitioners (*n* = 15)	12 (80.0)
Practice nurses (*n* = 16)	14 (93.3)

Participants (*n* = 149)	
Age (years), mean (SD)	56.8 (17.2)
Male sex, *n* (%)	77 (51.7)
BMI (kg/m^2^), mean (SD)	27.5 (5.2)
Smoking status, *n* (%)	
Current smoker	30 (20.1)
Stopped <1 year ago	7 (4.7)
Stopped ≥1 year ago	60 (40.3)
Never smoker	52 (34.9)
Previous pulmonologist visit (*n* = 146), *n* (%)	
No	95 (65.1)
Yes, <6 months ago	4 (2.7)
Yes, ≥6 months ago	47 (32.2)
Number of antibiotics/predniso(lo)ne courses in the previous year (*n* = 136), *n* (%)	
0	95 (69.9)
1	25 (18.4)
>1	16 (11.8)
Age of onset of respiratory symptoms (*n* = 137), median (IQR)	40.0 (11.0–59.0)
MRC (*n* = 128), *n* (%)	
0–2	116 (90.7)
>2	12 (9.3)
ACQ-5 (*n* = 134), *n* (%)	
<0.75	54 (40.3)
0.75–1.5	36 (26.9)
>1.5	44 (32.8)
CCQ, median (IQR)	
Total (*n* = 138)	1.0 (0.6–1.6)
Symptoms (*n* = 138)	1.6 (1.0–2.5)
Functional status (*n* = 139)	0.8 (0.3–1.5)
Mental (*n* = 139)	0.0 (0.0–0.5)
FEV_1_ (L)^a^, mean (SD)	2.6 (1.0)
FEV_1_ % predicted^a^, mean (SD)	79.1 (19.6)
FVC (L)^a^, mean (SD)	3.8 (1.1)
FEV_1_/FVC (%), mean (SD)	66.3 (12.4)
Reversibility testing performed, *n* (%)	90 (60.4)

*BMI* body mass index, *MRC* Medical Research Council dyspnoea scale, *ACQ* Asthma Control Questionnaire, *CCQ* COPD Clinical Questionnaire, *FEV*_*1*_ forced expiratory volume in 1 s, *FVC* forced vital capacity.

^a^Based on largest pre-bronchodilator value.

### Agreement on diagnosis

**Fig. 2:**
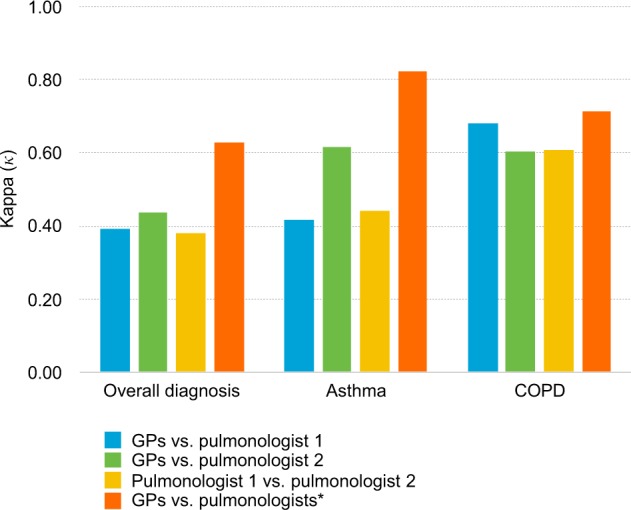
Agreement on diagnosis. Agreement on overall diagnosis, asthma and COPD between the GPs and pulmonologists (*n* = 140), between the pulmonologists (*n* = 141) and between the GPs and pulmonologists when only including cases on which the two pulmonologists agreed on diagnosis (*n* = 55). *Includes all cases on which the two pulmonologists agreed on diagnosis (*n* = 55).

The formulated diagnoses were reclassified into four categories: asthma, COPD, no signs for respiratory disease and other (which includes asthma/COPD overlap (ACO), restrictive disease, diagnosis unclear and other diagnosis). The overall observed agreement on diagnosis between GPs and pulmonologist 1 was 55.7% with *κ* 0.392 (95% CI 0.217−0.441). The overall agreement on diagnosis between GPs and pulmonologist 2 was the highest with an observed agreement of 59.3% and a moderate agreement according to Cohen’s kappa (*κ* 0.438, 95% CI 0.322−0.554). The overall observed agreement on diagnosis between the pulmonologists was 55.3% with *κ* 0.382 (95% CI 0.268−0.496) (Table 2 and Fig. 2).

**Table 2:** Agreement between GPs and pulmonologist 1 (a), GPs and pulmonologist 2 (b) and pulmonologists (c) on the presence of asthma, COPD, no respiratory disease or other diagnoses.

a					
	GP				
	Asthma	COPD	No disease	Other	Total
Pulm 1					
Asthma	18	2	0	5	25
COPD	0	33	0	14	47
No disease	3	1	8	6	18
Other	20	2	9	19	50
Total	41	38	17	44	140

b						
		GP				
		Asthma	COPD	No disease	Other	Total
Pulm 2						
Asthma		29	1	0	9	39
COPD		0	25	0	8	33
No disease		0	0	9	7	16
Other		12	12	8	20	52
Total		41	38	17	44	140

c						
		Pulm 1				
		Asthma	COPD	No disease	Other	Total
Pulm 2						
Asthma		18	1	0	6	25
COPD		2	29	0	17	48
No disease		2	1	8	7	18
Other		17	2	8	23	50
Total		39	33	16	53	141

*GP* general practitioner, *Pulm* pulmonologist, *COPD* chronic obstructive pulmonary disease.

Additional post hoc analysis showed an overall observed agreement on diagnosis between GPs and pulmonologists of 74.5% with a substantial agreement according to Cohen’s kappa (*κ* 0.627, 95% CI 0.480−0.774), when only cases on which the two pulmonologists agreed on the diagnosis of asthma, COPD or no respiratory disease were analysed (*n* = 55). A good agreement between GPs and pulmonologists was found for the diagnosis of asthma (*κ* 0.825, 95% CI 0.662−0.988) (Fig. 2 and Supplementary Table 1).

When looking at spirometry tests that met the ATS/ERS criteria only (*n* = 20), a substantial to good agreement on COPD diagnosis was found, whereas a moderate to substantial agreement was found in spirometry tests that did not meet the ATS/ERS criteria (*n* = 120) (Supplementary Table 2). Moreover, exclusion of respiratory disease tends to be more agreed on when the ATS/ERS criteria are met. A smaller difference is found for agreement on overall respiratory diagnosis; the agreement was moderate in tests that met the ATS/ERS criteria compared to a fair agreement (between GPs and pulmonologist 1 and between pulmonologists) and a moderate agreement (between GPs and pulmonologist 2) in tests that did not meet the ATS/ERS criteria.

### ATS/ERS criteria

Only 20 spirometry tests (13.4%) met the full set of ATS/ERS criteria (Table 3) (for the distribution of the individual practices, see Supplementary Fig. 1). The main reason for not meeting the criteria was poor compliance to the acceptability criteria, with the criteria on the peak expiratory flow (PEF) (‘good start of expiration’ and ‘reached peak with maximal effort’) being the least adhered to. The repeatability criteria were met in most of the spirometry tests, when assessing all spirometry tests (regardless of obtaining three acceptable curves). Of the 102 and 107 spirometry tests that did not meet the ATS/ERS criteria as assessed by lung function technicians 1 and 2 respectively, 22.5% and 20.6% of the spirometry tests did not adhere to only one of the acceptability criteria. When the criterion ‘exhalation ≥ 6 seconds’ was included in the analysis, which was met in 73.2% (lung function technician 1) and 67.1% (lung function technician 2) of the tests, only 16 (10.7%) spirometry tests met the ATS/ERS criteria. The proportion of spirometry tests that met the ATS/ERS criteria in the study group was not significantly different from the proportion of tests conducted prior to the study (11.1% vs. 13.4%, *p* value 0.804; Supplementary Table 3).

**Table 3:** Spirometry tests that did and did not meet the ATS/ERS acceptability and repeatability criteria (*n* = 149).

	LFT 1	LFT 2
ATS/ERS criteria met (acceptability and repeatability)	20 (13.4)	20 (13.4)
ATS/ERS criteria not met	102 (68.5)	107 (71.8)
ATS/ERS criteria not assessable	27 (18.1)	22 (14.8)
Acceptability (three acceptable curves)^a^	20 (13.4)	20 (13.4)
Good start of expiration (PEF reached quickly)	55 (36.9)	47 (31.5)
Reached peak with maximal effort	53 (35.6)	58 (38.9)
Smooth continuous exhalation	87 (58.4)	77 (51.7)
Good exhalation (no pinching)	94 (63.1)	88 (59.1)
No extra breaths being taken during manoeuvre	142 (95.3)	140 (94.0)
Plateau (≥1 s < 0.025 L change in volume)	101 (67.8)	90 (60.4)
Repeatability^b^	136 (91.3)	136 (91.3)
Difference between two largest values of FEV_1_ ≤ 0.150 L	146 (98.0)	146 (98.0)
Difference between two largest values of FVC ≤ 0.150 L	136 (91.3)	136 (91.3)
Acceptability (≥2 acceptable curves)	49 (32.9)	49 (32.9)

All values are *n* (%).

*ATS/ERS* American Thoracic Society/European Respiratory Society, *LFT* lung function technician.

^a^Duration is not used as a criterion for the three acceptable curves.

^b^Repeatability was assessed regardless of obtaining three acceptable curves.

### Quality and clinical usefulness

**Fig. 3:**
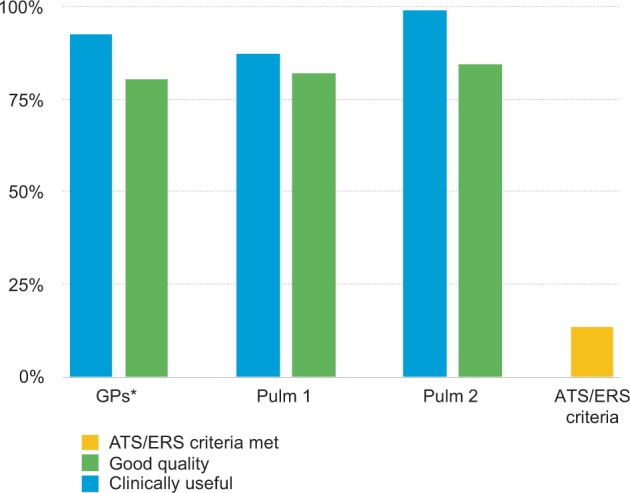
Clinical usefulness, quality of spirometry and ATS/ERS criteria. Clinical usefulness and quality of the spirometry tests (*n* = 149) as assessed by the GPs and pulmonologists and ATS/ERS criteria as assessed by the lung function technicians. GP general practitioner, Pulm pulmonologist, ATS/ERS American Thoracic Society/European Respiratory Society. *One assessment by the GPs was missing.

Overall, more than 80% of spirometry tests were assessed as good quality (GPs, 80.4%; pulmonologist 1, 81.9% and pulmonologist 2, 84.6%). Tests were rated as clinically useful in 92.5%, 87.5% and 99.3% of cases by the GPs, pulmonologist 1 and pulmonologist 2 respectively (Fig. 3, Supplementary Table 4).

### Annual number of spirometry tests

No correlation was found between the annual number of spirometry tests performed in the general practices and the proportion of spirometry tests on which the GPs and pulmonologist 1 agreed on diagnosis (*n* = 12; Spearman’s correlation coefficient −0.281, *p* value 0.377). However, a negative correlation was found between the annual number of tests and the proportion of tests on which the GPs and pulmonologist 2 agreed on diagnosis (*n* = 12; Spearman’s correlation coefficient −0.635, *P* value 0.027). No correlation was found between the annual number of spirometry tests performed in the general practices and the proportion of spirometry tests that met the ATS/ERS criteria (*n* = 12; Spearman’s correlation coefficient 0.089, *P* value 0.784).

### Treatment advice

GPs and pulmonologists 1 and 2 formulated the advice to continue current treatment 58, 57 and 51 times respectively. In 23 and 24 cases the GPs and pulmonologists 1 and 2 agreed on this advice. The pulmonologists recommended to stop current medication more often than GPs (38 and 17 times vs. 2 times), whereas the GPs recommended an increase in dose of current medication more often (12 vs. 1 and 3 times). Both GPs and pulmonologists 1 and 2 recommended smoking cessation, more physical exercise and discussion of diet in more than 24 patients (range 24–68). Review of medication adherence and inhaler technique was frequently recommended by GP and pulmonologist 2 (26 and 32 times respectively), but only twice by pulmonologist 1. Referral to a pulmonologist was only recommended 6 times by the GPs and 34 and 45 times by pulmonologists 1 and 2 respectively.

## Discussion

In this study, we evaluated the quality of spirometry by agreement on respiratory diagnosis between GPs and pulmonologists in a real-life setting. We found a low agreement on respiratory diagnosis between GPs and pulmonologists and also between the pulmonologists. Agreement on COPD was the highest, followed by asthma. When only including cases on which pulmonologists agreed on diagnosis, a much higher agreement on diagnosis was found between GPs and pulmonologists. A remarkably large difference was found in the amount of clinically useful spirometry test as assessed by the pulmonologists and GPs (87%) and spirometry tests that met the acceptability and repeatability criteria as defined by ATS/ERS (13%).

Agreement on diagnosis based on spirometry has been evaluated in two previously published studies^13,14^. However, those studies were not comparable to the present study. One study compared the assessment of standardised case descriptions by GPs to a golden standard (consensus within an expert panel) and offered all participating GPs a study-specific spirometry training^14^. The second study only included patients with (a suspicion of) COPD and provided spirometry training for the participating GPs as well^13^. No studies were found that evaluated agreement on diagnosis between GPs and pulmonologists without providing study-specific spirometry training and using real patients that were included consecutively.

Our finding of a large proportion of tests being clinically useful is consistent with the study by Landman et al.^12^. In contrast to that study, in which clinical usefulness of spirometry tests was based on the opinion of lung function technicians, clinical usefulness in the present study was based on the opinion of GPs and pulmonologists. The current approach may be a more representative estimate of clinical usefulness, as physicians are the ones who formulate a diagnosis and treatment advice in real-world practice.

Adherence to the ATS/ERS criteria is found to be higher in other studies conducted in a primary care setting (32−40%)^11,12,15^. This difference could be partly explained by the requirement of only two acceptable flow-volume curves in one of those studies^12^, as their results are comparable to the 33% meeting the ATS/ERS acceptability criteria in two curves found in the current study. Furthermore, participation of the involved practices in a regional working agreement with a hospital in the previous studies could have influenced adherence to ATS/ERS criteria, as regular spirometry training and support was part of this agreement^12,15^. In addition, intensive study-specific spirometry training offered to healthcare professionals administering the tests may explain the difference in results^15^. Only one publication was found on adherence to ATS/ERS criteria in secondary care, showing that 41% of the assessed spirometry tests met the ATS/ERS criteria^16^.

‘Duration of exhalation of a minimum of six seconds’ and ‘reaching a volume/time plateau for longer than one second’ have been identified as the ATS/ERS criteria the least adhered to in spirometry testing in primary care^11,12^. This could result in underestimation of FVC and thus, an overestimation of the FEV_1_/FVC ratio. As a consequence, airway obstruction may be underestimated. In contrast to those previous studies, we found the criteria ‘good start of expiration’ and ‘reached peak with maximal effort’, to be the criteria the least adhered to. A suboptimal PEF could lead to an underestimation and even an overestimation of FEV_1_, which may result in an under- or overestimation of airway obstruction. One of the reasons for poor scores on the PEF-related criteria might be the fact that the software used by general practices did not recognise a poor PEF in most of the cases. Although nearly all participating practice nurses followed a spirometry course, they might rely too much on the support of the software. In addition, the problem of underestimation of FVC is highlighted in current education programmes, which might have resulted in improvement of the related criteria. To improve diagnostic accuracy, future spirometry training should focus more on the importance of a good start and peak. Also, the PEF should receive more attention in the development of spirometry software programmes.

Besides the poor compliance to PEF-related criteria, one out of six spirometry tests was not assessable at all. This was mostly due to wrong spirometry settings (e.g. start of spirometry not visible in curve), an improperly maintained flow sensor (e.g. no plateau reached after 15 s of exhalation) or wrong printing settings (e.g. composite curves). We have included these spirometry tests in the analyses, as these are the spirometry tests that are used by the GP to provide clinical advice and therewith, reflect daily practice.

A limitation of this study was that the assessment of diagnosis by GPs could have been influenced by the fact that GPs possibly know study participants from previous consultations. For example, it is known that airway obstruction is not always found by spirometry testing in mild to moderate asthma patients^3^. In these patients, assessment of diagnosis was based on the completed questionnaires and an inconclusive spirometry. GPs could have formulated the diagnosis asthma if they knew the study participant from previous consultations, whereas the pulmonologist assessed anonymised data and would not formulate the diagnosis asthma. We estimate this influence to be small, as both GPs and pulmonologists had access to the completed questionnaires, which included respiratory history filled in by the patient and by the practice nurse. Furthermore, only consecutive spirometry tests from a whole general practice were included in this study. In the Dutch primary care setting, often one out of two to four GPs in a practice is trained and reviews all spirometry tests, including those from patients of GP colleagues.

In total, one out of three spirometry tests combined with the structured clinical data was assigned the diagnosis ‘unclear’ by the pulmonologists. No additional investigations were performed in those patients to find a diagnosis, as this was not the aim of the study. However, most of the spirometry tests were assessed as clinically useful (74.5% and 100% of the tests that had been assigned the diagnosis ‘unclear’ by pulmonologists 1 and 2 respectively). Furthermore, the diagnosis ‘unclear’ was not expected to be assigned less often when the pulmonologists would have performed live assessments, as in previous research good concordance was found between live assessment and paper assessment (*κ* 0.82)^17,18^. This reflects the difficulties in formulating a diagnosis based on the diagnostic facilities available in primary care respiratory medicine, which would warrant referral in one third of patients for further assessment^19,20^.

In this study, the respiratory diagnosis as formulated by the pulmonologist was supposed to be the gold standard. To ensure the gold standard was represented thoroughly, we have chosen to include two pulmonologists, as we did for the lung function technicians. The agreement on ATS/ERS criteria between the lung function technicians was good (*κ* 0.67 before consensus meetings, *κ* 0.81 after consensus meetings). In contrast, the agreement on diagnosis between the pulmonologists was only fair according to kappa (*κ* 0.38). In COPD the agreement was highest, but not as high as could be expected based on the fact that COPD is a diagnosis strictly defined by spirometry findings. Variation in diagnosis in respiratory medicine has been found before. In asthma for example, the diagnosis is the result of a complex assessment because it is less dependent on spirometry, resulting in higher variation between physicians and relevant misdiagnosis^19^. We performed a post hoc analysis using only cases on which the two pulmonologists agreed on the diagnosis, showing a substantial agreement on diagnosis between GPs and pulmonologists according to Cohen’s kappa. As numbers are small (*n* = 55), these results should be confirmed in larger studies. For future evaluation of diagnostic accuracy, the gold standard might need to be extended with an expert panel.

The advantages of performing spirometry in primary care are large, but sufficient quality should be assured. This real-life study demonstrated that agreement on respiratory diagnosis between GPs and pulmonologists is relatively low, as is the agreement between pulmonologists, based on spirometry, patient history and symptoms. When assessing the group of patients in which the two pulmonologists both agreed on the diagnosis, agreement between pulmonologists and GPs was much higher. Only a few spirometry tests met ATS/ERS criteria, but clinical usefulness was very high as rated by both GPs and pulmonologists. This suggests that meeting the ATS/ERS criteria may not be required for providing a diagnosis, when physicians are offered spirometry results and questionnaires on patient history and symptoms. However, it is unclear if quality of spirometry based on the formulated diagnosis is higher when the ATS/ERS criteria are met. Therefore, further research should focus on evaluating the influence of meeting ATS/ERS criteria on clinical decision making in a real-life setting.

## Methods

### Study design and participants

This prospective observational study was conducted in general practices in the area of Zwolle, the Netherlands. All spirometry-performing general practices interested in participating were eligible for inclusion. Practices in the area of Zwolle were invited to participate by phone or e-mail. Effort was put into including regular practices in the study, also those that do not regularly take part in respiratory medical research. All participants aged 18 years and over, who underwent spirometry as part of usual care indicated by their GP, were eligible for inclusion in this study and were asked to participate. All participating GPs were asked to include ten spirometry tests from ten consecutive patients performed in their general practice irrespective of the eventual diagnosis, to ensure objective inclusion of spirometry tests. In addition, practices were asked to provide three spirometry tests performed one, two and three months before the start of study. All general practices performed spirometry tests according to the ATS/ERS guidelines^7^. Administration of bronchodilators to perform reversibility testing was done only when indicated by the GP. The medical ethics committee of the University Medical Center Groningen (UMCG) deemed that formal medical ethical approval was not required, as this study did not fall under the Dutch Medical Research Involving Human Subjects Act. This study is reported in accordance with the ‘Strengthening the reporting of observational studies in epidemiology’ (STROBE) Statement^21^.

### Study procedures

Written informed consent was obtained from participants before starting any study-specific procedures. Participants were able to withdraw from the study during their participation, without giving a reason. Participants were asked to complete a medical history questionnaire based on the Dutch asthma and COPD guidelines (Supplementary Table 5)^4,5^, assessing gender, age, BMI, respiratory medication use, smoking status, comorbidities, age of onset of respiratory symptoms, family respiratory history, profession, bronchial hyperresponsiveness and whether or not a patient visits a pulmonologist on a regular basis. Furthermore, the following questionnaires were completed: the Medical Research Council (MRC) dyspnoea scale with higher scores indicating more impact of breathlessness on daily activities^22^, the Asthma Control Questionnaire (ACQ) measuring asthma control (five items) with higher scores indicating worse asthma control^23^, and the Clinical COPD Questionnaire (CCQ) assessing health status in COPD patients (ten items) with higher scores indicating worse health status^24^. After completion of the questionnaires, spirometry was performed. The practice nurse was asked to select three pre-bronchodilator curves and, when performed, three post-bronchodilator curves. Participating general practices were asked to fill in questions about the type of spirometer, frequency of calibration of the spirometer, the annual number of spirometry tests performed, number of operators in the practice and the date of last participation in a spirometry education programme.

### Assessment of spirometry tests

GPs formulated a diagnosis and treatment advice for all included patients of their general practice, based on the completed questionnaires and spirometry test results printed on paper including post-bronchodilator curves when performed. In addition, GPs assessed the spirometry tests results on quality (good, moderate or poor) and clinical usefulness (useful or not useful). In this case, clinical usefulness means that the quality of spirometry is considered sufficient to make clinical decisions. An example of the assessment form is provided in Supplementary Fig. 2.

Subsequently, the spirometry test results on paper supplied with the completed questionnaires on paper were sent to two pulmonologists from the Isala Hospital in Zwolle. All spirometry test results had the same lay-out and patient identifiers were removed from the spirometry test results before sending. The pulmonologists evaluated the spirometry test results on the same criteria as the GPs did. The pulmonologists were blinded for the assessment of the GP and for each other’s assessments.

Two lung function technicians from the Pulmonary Laboratory in the Isala Hospital assessed the spirometry test results (including the spirometry tests performed prior to the study) on acceptability and repeatability as defined by the ATS/ERS criteria, which are specified in Box 1 ^7,25^. According to the ATS/ERS criteria, a subject should try to exhale for at least 6 s. However, some healthy adults are able to empty their lungs within 6 s. The lung function technicians were not able to assess if subjects tried to exhale for at least 6 s, because they did not conduct the test themselves. Therefore, the criterion ‘duration exhalation ≥ 6 s’ has not been included in our main analysis, meaning that reaching a plateau (≥1 s < 0.025 L change in volume) within 6 s was sufficient to meet the end of test criteria. A sensitivity analysis in which the duration criterion is included has been performed. A spirometry test was considered ‘not assessable’ when the lung function technicians were not able to assess one or more ATS/ERS criteria (e.g. because of wrong software settings). In addition, the lung function technicians assessed the number of acceptable curves according to the ATS/ER criteria. After completing data collection, consensus meetings were held to discuss disagreement in assessment between the two lung function technicians. All tests with disagreement on acceptability or repeatability (i.e. one lung function technician assessed the spirometry as acceptable or repeatable, while the other lung function technician did not) were discussed. Furthermore, tests that were considered as not assessable by one lung function technician were discussed when acceptability or repeatability could still be met by discussing the assessments. In all cases, discussion was sufficient to resolve the disagreement between the technicians. Finally, tests with disagreement on the number of acceptable curves according to the lung function technician’s opinion were discussed.

Box 1 Criteria used as assessment tool for lung function technicians, based on the ATS/ERS criteria as described by Miller et al.^7^

**Table:** 

Acceptability criteria^a^
*Flow−volume curve*
1. Good start of expiration—PEF reached quickly (extrapolated volume <5% or FVC < 0.15 L)
2. Reached peak with maximal effort
3. Smooth continuous exhalation (no cough during the first second)
4. Good exhalation (no glottis closure, pinched exhalation or hesitation)
5. No extra breaths being taken during the manoeuvre
*Volume−time curve*
6. Duration exhalation ≥ 6 s^b^
7. Plateau (≥1s < 0.025 L change in volume)

Repeatability criteria^c^
1. Difference between two largest values of FVC < 0.150 L
2. Difference between two largest values of FEV_1_ < 0.150 L
^a^At least three acceptable curves have to be obtained.
^b^Duration is not used as a criterion for three acceptable curves in this study.
^c^Apply repeatability criteria after three acceptable curves have been obtained.

### Sample size

The sample size was calculated using the method of Cohen’s Kappa as described by Cantor^26^. The calculation was made by estimating kappa with a 95% confidence interval (95%CI) of ±0.15. No assumptions were made concerning the size of kappa; therefore, the minimal possible kappa was used. In this method the variable *Q*, associated with kappa, was used to calculate the sample size. Based on a study by Schneider et al.^15^, the diagnosis asthma is expected to be made in 56% of cases by the GP and in 41% of cases by the pulmonologist. The minimum kappa was associated with *Q* = 0.852 ^26^. As a result, 146 participants were needed.

### Statistical analysis

Study data were collected and managed using REDCap (Research Electronic Data Capture)^27^. The statistical analysis was performed using the statistics software package IBM SPSS Statistics for Windows, version 25.0 (IBM Corp., Armonk, NY). Participant and general practice characteristics were summarised using descriptive statistics and frequency distributions. Baseline characteristics are shown as mean ± standard deviation or, in case of non-normally distributed data, median and interquartile range (IQR). The primary outcome of this study was agreement between the GP and pulmonologists on the formulated diagnosis. Agreement on diagnosis is not expected in spirometry tests of poor quality or tests that are clinically useless. Therefore, spirometry tests that were assessed as being clinically useless by both pulmonologists were excluded from the analysis. Also, tests that were assessed as being of poor quality by both pulmonologists or as moderate quality by one pulmonologist and poor quality by the other pulmonologist were excluded from the analysis. Agreement on diagnosis is expressed as observed agreement and Cohen’s kappa (*κ*). Observed agreement is defined as the number of tests on which the raters agree with each other divided by the total number of tests (*a* + *d*/*N*). Agreement with Cohen’s kappa is interpreted as described by Landis and Koch^28^: *κ* > 0.81 is considered a good agreement, *κ* > 0.61 a substantial agreement, *κ* > 0.41 a moderate agreement and *κ* > 0.21 a fair agreement. In addition, a post hoc analysis was performed on agreement on diagnosis between the GPs and pulmonologists, only including cases on which the two pulmonologists agreed on diagnosis of asthma, COPD or no respiratory disease. This agreement is expressed as observed agreement and Cohen’s kappa.

Prespecified secondary outcomes included (1) interrater agreement on diagnosis between the two pulmonologists expressed as observed agreement and Cohen’s kappa, (2) proportion spirometry tests that met and did not meet the ATS/ERS criteria, (3) proportion of clinically useful spirometry tests and proportion of spirometry tests of good quality based on the opinion of GPs and pulmonologists, (4) agreement on diagnosis in spirometry tests that did and did not meet ATS/ERS criteria expressed as Cohen’s kappa, (5) the correlation between the proportion of spirometry tests on which the GP and pulmonologist agreed on diagnosis and the yearly number of spirometry tests performed by the GP (Spearman’s correlation coefficient), (6) the correlation between the proportion of spirometry tests that met the ATS/ERS criteria and the yearly number of spirometry tests performed by the GP (Spearman’s correlation coefficient), (7) whether the Hawthorne effect (change of behaviour in response to the awareness of participation in a trial) was present by comparing the proportion of spirometry tests that met the ATS/ERS criteria before the start of the study and during the study and (8) frequencies of the formulated treatment advices.

### Reporting summary

Further information on research design is available in the Nature Research Reporting Summary linked to this article.

## Supplementary information

Supplementary Information

Reporting Summary

## Data Availability

The data that support the findings of this study are available from the corresponding author upon reasonable request. All data provided will be anonymized.
