# Supplementary material for: Quality of spirometry and related diagnosis in primary care with a focus on clinical use
Source: NPJ Prim Care Respir Med. 2020 May 15;30:22. doi: 10.1038/s41533-020-0177-z (PMC7229174; doi:10.1038/s41533-020-0177-z)
Supplement: Supplementary file 1 — Supplementary Information [file 41533_2020_177_MOESM1_ESM.pdf]

SUPPLEMENTARY INFORMATION

Supplementary Table 1: Agreement on diagnosis between the GPs and pulmonologists (a,b), between the pulmonologists (c) and between GPs and pulmonologists when only including cases on which the two pulmonologists agreed on diagnosis (d).

	**a) GP x Pulm1 (n=140)**	**b) GP x Pulm2 (n=140)**	**c) Pulm1 x Pulm2 (n=141)**	**d) Pulm x GP (n=55)**
Overall OA (%)	55.7	59.3	55.3	74.5
Overall	0.392 [0.217; 0.441]	0.438* [0.322; 0.554]	0.382 [0.268; 0.496]	0.627** [0.480; 0.774]
Asthma	0.416* [0.247; 0.585]	0.615** [0.470; 0.760]	0.442* [0.273; 0.611]	0.825*** [0.662; 0.988]
COPD	0.681** [0.550; 0.812]	0.604* [0.451; 0.757]	0.607* [0.466; 0.748]	0.712** [0.532; 0.892]
No disease	0.380 [0.155; 0.605]	0.485* [0.262; 0.708]	0.398 [0.173; 0.623]	0.740** [0.464; 1.016]
Other	0.105 [-0.062; 0.272]	0.116 [-0.051; 0.283]	0.129 [-0.038; 0.296]	-

*Values are κ (95% CI) unless otherwise stated. GP, General Practitioner; Pulm, pulmonologist; COPD, Chronic Obstructive Pulmonary Disease; OA, observed agreement (a+d / total); κ, Cohen's kappa; *κ > 0.41 (moderate agreement), ** > 0.61 (substantial agreement), *** > 0.81 (good agreement).*

Supplementary Table 2: Agreement between pulmonologists and between GPs and pulmonologists in spirometry tests that meet ATS/ERS criteria and in spirometry tests that did not meet ATS/ERS criteria

	**GP x Pulm1 (n=140)**		**GP x Pulm2 (n=140)**		**Pulm1 x Pulm2 (n=141)**	
	ATS/ERS criteria met (n=20)	ATS/ERS criteria not met (n=120)	ATS/ERS criteria met (n=20)	ATS/ERS criteria not met (n=120)	ATS/ERS criteria met (n=20)	ATS/ERS criteria not met (n=121)
Agreement (%)	60.0	55.0	60.0	59.1	60.0	54.5
Overall (κ, 95% CI)	0.443* [0.139; 0.749]	0.382 [0.260; 0.504]	0.414* [0.116; 0.712]	0.437* [0.314; 0.560]	0.448* [0.158; 0.738]	0.363 [0.240; 0.486]
Asthma (κ)	0.348	0.422*	0.510*	0.627**	0.327	0.450*
COPD (κ)	0.857***	0.655**	0.688**	0.592*	0.857***	0.572*
No disease (κ)	0.459*	0.370	0.643**	0.467*	0.773**	0.334
Other (κ)	0.205	0.090	-0.013	0.140	0.028	0.132

*GP, General Practitioner; Pulm, pulmonologist; ATS/ERS, American Thoracic Society / European Respiratory Society; COPD, Chronic Obstructive Pulmonary Disease; κ, Cohen's kappa; *κ > 0.41 (moderate agreement), ** > 0.61 (substantial agreement), ***>0.81 good agreement.*

Supplementary Figure 1: ATS/ERS outcome per practice

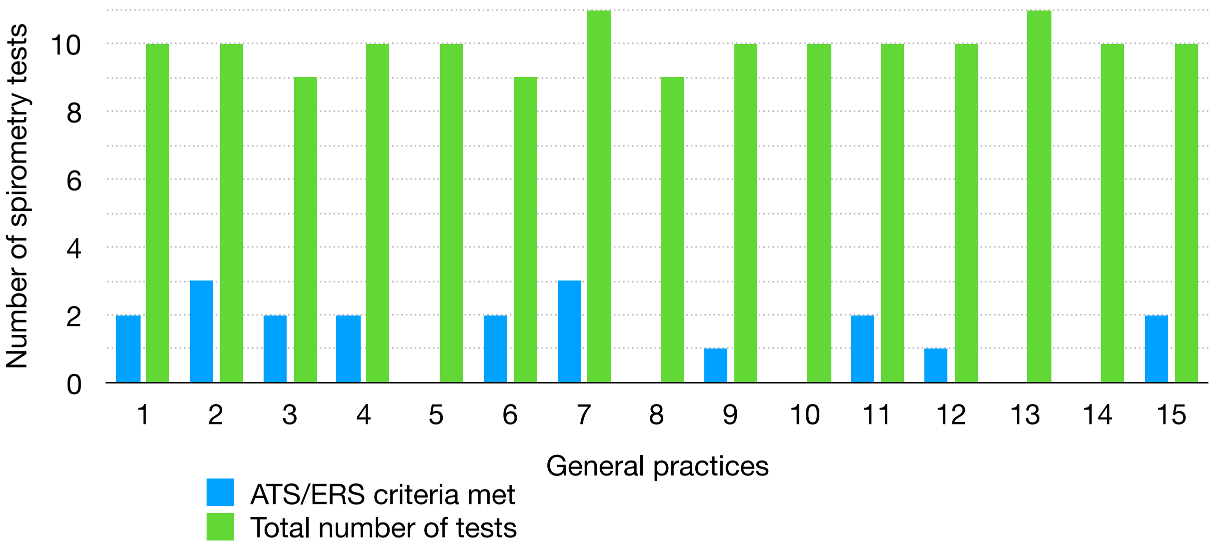

The number of patients contributing data from each practice and the number of spirometry tests meeting ATS/ERS criteria in each practice

Supplementary Table 3: Participant characteristics and spirometry tests that met ATS/ERS criteria of study and pre-study participants

	**Study group (n=149)**	**Pre-study group (n=45)**	**P-value**
Age (years)	56.8 (17.2)	54.1 (17.2)	0.361
Male sex, *n (%)*	77 (51.7)	22 (48.9)	0.865*
BMI (kg/m^2)	27.5 (5.2)	28.1 (4.7)	0.467
FEV_1 (L)	2.6 (1.0)	2.7 (0.9)	0.523
FEV_1 % predicted	79.1 (19.6)	81.8 (16.4)	0.412
FVC (L)	3.8 (1.1)	3.8 (1.0)	0.934
FEV_1/FVC (%)	66.3 (12.4)	69.8 (10.1)	0.057
ATS/ERS criteria met	20 (13.4)	5 (11.1)	0.804*
ATS/ERS criteria met including 'duration exhalation ≥ 6s'	16 (10.7)	4 (8.9)	1.000*

BMI, Body Mass Index; FEV_1, Forced Expiratory Volume in 1 second; FVC, Forced Vital Capacity; ATS/ERS, American Thoracic Society / European Respiratory Society.
All values are mean (SD), unless stated otherwise. Independent t-test is used unless stated otherwise.
**Fisher's exact test.*

Supplementary Table 4: Quality and clinical usefulness assessments by the GPs and pulmonologists

	GPs (n=148)	Pulm 1 (n=149)	Pulm 2 (n=149)
**Quality assessment**			
Good quality	119 (80.4)	122 (81.9)	126 (84.6)
Moderate quality	22 (14.9)	16 (10.7)	22 (14.8)
Poor quality	7 (4.7)	11 (7.4)	1 (0.7)
**Clinical usefulness assessment**			
Clinically useful	135 (92.5)	130 (87.2)	148 (99.3)
Not clinically useful	11 (7.5)	19 (12.8)	1 (0.7)

GP, General Practitioner; Pulm, Pulmonologist.

Supplementary Table 5: Topics assessed in the medical history questionnaire completed by participants and practice nurses

**Completed by participant**
Age
Sex
Height
Weight
Previous pulmonologist visit
Medication<br>Use<br>Side effects<br>Compliance
Smoking status
Use of antibiotics or predniso(lo)ne in previous year
Age at onset of respiratory symptoms
History of respiratory and allergic diseases
Family respiratory history
Occupation
Triggers for respiratory symptoms
MRC
CCQ
ACQ
**Completed by practice nurse**
Known respiratory diseases
Number of attempted blows

Supplementary Figure 2: Spirometry assessment form for GPs and pulmonologists

What disease would you diagnose, based on the provided data?

☐ Asthma
☐ COPD
☐ Asthma/COPD overlap (ACO)
☐ Suspicion of restrictive disease
☐ No signs of airway obstruction, asthma or COPD
☐ Diagnosis unclear
☐ Other (specify), …

Which treatment would you advice to start/add? (multiple answers are allowed)

☐ Continue current treatment
☐ Increase the dose of current medication
☐ Decrease the dose of current medication
☐ Stop current medication
☐ Change way of administration of current medication
☐ Short Acting Beta-2 Agonist (SABA) (as needed)
☐ Long Acting Beta Agonist (LABA)
☐ Short Acting Muscarinic Antagonist (SAMA)
☐ Long Acting Muscarinic Antagonist (LAMA)
☐ Low dose inhaled corticosteroids (ICS)
☐ Moderate dose ICS
☐ High dose ICS
☐ Leukotriene Receptor Antagonist (LTRA)
☐ ICS + LABA
☐ Maintenance dose of oral corticosteroids
☐ Course of oral corticosteroids (high dose)
☐ Recommend to stop smoking
☐ Recommend more physical exercise
☐ Discuss diet (obesity)
☐ Review medication adherence/inhaler technique
☐ Discuss anxiety and/or a possible depression
☐ Refer to pulmonologist
☐ Other (specify), …

What is your opinion on the quality of the spirometry test?

☐ Good quality
☐ Moderate quality
☐ Poor quality

What is your opinion on the clinical usefulness of the spirometry test?

☐ Clinically not useful
☐ Clinically useful
